# Enhanced Photocatalytic Degradation Activity Using the V_2_O_5_/RGO Composite

**DOI:** 10.3390/nano13020338

**Published:** 2023-01-13

**Authors:** Anuja A. Yadav, Yuvaraj M. Hunge, Seok-Won Kang, Akira Fujishima, Chiaki Terashima

**Affiliations:** 1 Department of Automotive Engineering, Yeungnam University, 280 Daehak-ro, Gyeongsan 38541, Republic of Korea; 2 Research Center for Space System Innovation, Research Institute for Science and Technology (RIST), Tokyo University of Science, 2641 Yamazaki, Noda, Chiba 278-8510, Japan

**Keywords:** photocatalysis, methylene blue, V_2_O_5_/RGO

## Abstract

Semiconductor-based photocatalyst materials played an important role in the degradation of organic compounds in recent years. Photocatalysis is a simple, cost-effective, and environmentally friendly process for degrading organic compounds. In this work, vanadium pentoxide (V_2_O_5_) and V_2_O_5_/RGO (reduced graphene oxide) composite were synthesized by a hydrothermal method. The prepared samples were characterized by X-ray diffraction (XRD), X-ray photoelectron spectroscopy (XPS), scanning electron microscopy (SEM), Raman spectroscopy, and UV-Vis spectroscopic analysis, etc. Raman analysis shows the occurrence of RGO characteristic peaks in the composite and different vibrational modes of V_2_O_5_. The band gap of flake-shaped V_2_O_5_ is reduced and its light absorption capacity is enhanced by making its composite with RGO. The photocatalytic degradation of methylene blue (MB) was studied using both V_2_O_5_ and V_2_O_5_/RGO composite photocatalyst materials. The V_2_O_5_/RGO composite exhibits a superior photocatalytic performance to V_2_O_5_. Both catalyst and light play an important role in the degradation process.

## 1. Introduction

In the present situation, environmental pollution is a major problem in the world, causing global damage to the life on the earth. Among the different types of environmental pollutions, such as water, soil, and air pollution, etc., water pollution has a major impact on aquatic life and living organisms. Water pollution is caused by the release of hazardous organic compounds, such as dyes, acids, and antibiotics, etc., from textile, chemical, and pharmaceutical factories into potable water bodies, such as rivers, lakes, and ponds, etc. Most of the organic compounds are carcinogenic in nature. In addition, water pollution directly leads to soil pollution, which directly or indirectly affects day-to-day life [1,2]. From the textile industries, synthetic colour dyes were released during the textile wash mix very well with water in comparison with the chemicals and reagents, so the mix of effluents has toxic properties. Therefore, it is essential to treat industrial effluents before discharging them into the environment. Due to the concern that the toxicity of the effluents has long-lasting effects on the ecosystem [3,4], it is, therefore, important to remove or treat industrial discharge by efficient and effective water treatment methods.

To date, different water treatment methods have been employed, such as biodegradation, coagulation, adsorption, and photocatalysis, etc., for the removal of organic impurities [5]. Among these methods, the photocatalysis method is reliable for the removal of toxic pollutants, or the conversion of toxic pollutants to less hazardous pollutants. This method has many advantages, e.g., it is environmentally friendly, there is no yield of secondary pollutants, it is cost-effective, and the catalyst is reusable. The photocatalytic activity of photocatalyst materials is depends upon the spectral response, the light absorption capacity and the rate of generation of charge carriers, i.e., electrons and holes [6,7]. Efficient photocatalysts should fulfill different requirements, such as good optical response, tunable band gap energy, good photo- and chemical stability, and high affinity for light, etc. [8].

In recent decades, metal-oxide-based semiconductors and their composites have been used for the degradation of organic pollutants, such as dyes, acids, and antibiotics, etc. [9]. TiO_2_ and ZnO semiconductor materials have been most commonly used by the research community from the last few decades, due to their easy preparation, good photo stabilities, and activities, but the major concerns raised with these materials are their wide band gap abilities and limited spectral responses, i.e., they absorb only ultraviolet light, leading to poor photocatalytic efficiency, etc. [10]. However, despite outstanding success in this field during the past few decades, but there are still major challenges to developing efficient, cost-effective, and robust materials, which are able to absorb visible light with good carrier conductivity, band positions, and stability, during photochemical reactions [11].

Several visible active semiconductor catalysts, such as Fe_2_O_3_, WO_3_, V_2_O_5_, etc., have been used for photocatalytic applications. However, among the different visible light active materials, V_2_O_5_ has received more attention due to its outstanding properties, such as strong absorption in the visible region, possession of strong photocatalytic activity, and good solar-to-hydrogen conversion efficiency [12,13]. The band gap value of V_2_O_5_ lies in the visible region, without the addition of any dopant or making heterojunctions, etc. In order to improve the photocatalytic performance, different strategies have been employed, such as coupling of V_2_O_5_ with other semiconductor materials, such as BiVO_4_, ZnO, GNS-V_2_O_5_–TiO_2_, etc. [14]. However, for the improvement of photocatalytic performance, carbonaceous materials are typically used because they are low in cost, environmentally friendly, and can be mass-produced. Reduced graphene oxide (RGO) has been widely used as a supporting material for enhancing charge transport properties, organic compound adsorption and light absorption capabilities, due to its outstanding electrical conductivity, large surface area, and good optical properties, with increased photogenerated charge carrier mobility in the photocatalytic process and tunable band gap [15]. V_2_O_5_ has been used for different applications, such as lithium-ion batteries, supercapacitors, photocatalysis, and water-splitting reactions, etc. Chauhan et al. synthesized V_2_O_5_-rGO nanocomposite and used it to study the photocatalytic degradation of rhodamine B [16]. Sharma et al. prepared V_2_O_5_/GO nanocomposite by a hydrothermal route and studied MB degradation and photoelectrochemical water splitting [11]. Therefore, V_2_O_5_/RGO composite may provide a new generation of material with excellent photocatalytic activity.

In the present work, a hydrothermal method was used to synthesize V_2_O_5_ and V_2_O_5_/RGO composite, in order to study the photocatalytic degradation of methylene blue (MB) dye. The bonds formed among the V-O-C are due to unpaired electrons in RGO and extend the visible light absorption capacity of the V_2_O_5_ catalyst. Additionally, the role of V^5+^ in pure V_2_O_5_ in absorption and photocatalytic activity is discussed, along with the role of RGO in the V_2_O_5_/RGO composite. In view of the above-mentioned facts, it is established that both V_2_O_5_ and RGO are good photocatalysts. Hence, composites of these two materials can be a good starting point for enhancing photocatalytic efficiency.

## 2. Experimental Details

### 2.1. Synthesis of V_2_O_5_

For the preparation of vanadium oxide (V_2_O_5_), a hydrothermal method was used. For the preparation of V_2_O_5_, ammonium metavanadate (NH_4_VO_3_) (Sigma Aldrich, St. Louis, MO, USA) and polyethylene glycol (Sigma Aldrich) were added to 40 mL of deionized water. The mixture was stirred until the solution was completely dissolved. The pH of the solution was adjusted with hydrochloric acid (HCl) and stirred continuously. This solution was then transferred to a Teflon-lined autoclave, the hydrothermal reactor was tightened and kept in a furnace at 150 °C for 12 h. The solution was then centrifuged and washed with DI water and ethanol several times. The V_2_O_5_ powder was dried in an oven.

### 2.2. Synthesis of RGO

The synthesis of graphene oxide (GO) was carried out using the Hummers method [17]. The process for the reduction of GO into RGO is described by Chauhan et al. [16,18].

### 2.3. Synthesis of V_2_O_5_/RGO Composite

V_2_O_5_ precursor solution was prepared as described in Section 2.1. To this precursor solution of V_2_O_5,_ an appropriate amount of RGO was added. The solution was stirred vigorously for 2 h at room temperature, and was then transferred to a 50 mL Teflon-lined autoclave (Techinstro, Nagpur, India) and kept in a furnace at 150 °C for 12 h. The autoclave was left to cool to room temperature, and the prepared V_2_O_5_/RGO composite was centrifuged, washed several timed with deionized water and ethanol, and dried.

### 2.4. Photocatalytic Degradation Experimental Details

The photocatalytic properties of V_2_O_5_ and V_2_O_5_/RGO composite for MB degradation were investigated. For this investigation of photocatalytic properties, an Xe-lamp was used as the source of illumination. A concentration of 0.025 mM MB (100 mL) and 25 mg of catalyst was used during each experiment. Before starting the photocatalytic experiment, i.e., in dark conditions, the reaction mixture of MB (100 mL) and 25 mg catalyst was stirred for 30 min to attain adsorption–desorption equilibrium. The reaction mixture was then exposed to the light source to start the photocatalytic reaction. After a specific interval of time, 3 mL of reaction solution was taken and centrifuged to remove traces of the catalyst. Using the UV-Vis spectrophotometer (Shimadzu: UV-1800, Kyoto, Japan), the change in concentration of MB was measured. In addition, a COD study was conducted to confirm the mineralization of MB dye. Details of the COD measurement procedure can be found in our previously published work [19].

## 3. Results and Discussion

**Figure 1:**
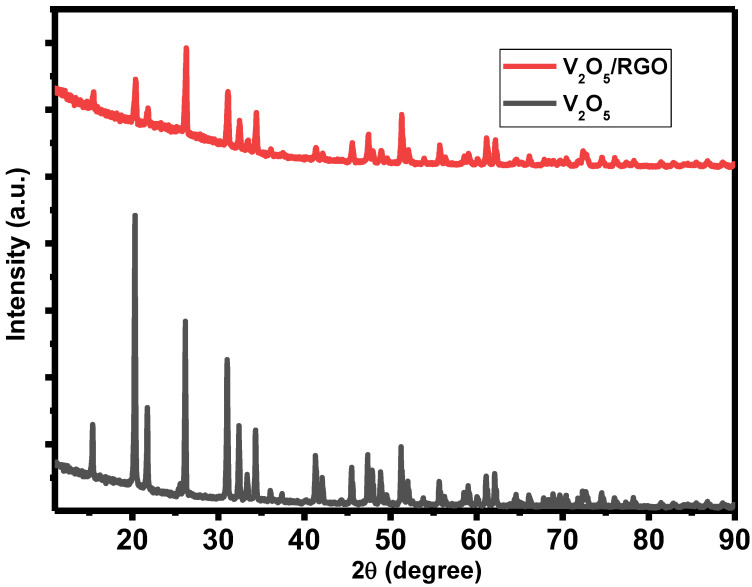
X-ray diffraction patterns of V_2_O_5_ and V_2_O_5_/RGO composite.

To investigate the crystal structure and phase formation of V_2_O_5_ and V_2_O_5_/RGO composite, X-ray diffraction characterization was performed and the results are presented in Figure 1. From the XRD pattern, the polycrystalline nature of the prepared catalyst materials is observed. For V_2_O_5_, major diffraction peaks are found at 2θ = 20.37°, 21.83°, 26.26°, and 31.08°, which correspond to (001), (101), (110), and (301) planes, respectively. All the diffraction peaks of V_2_O_5_ match well with the JCPDS card no. 41–1426 and confirm that the orthorhombic crystal structure is without any impurities [20]. In the V_2_O_5_/RGO composite, an RGO peak is not observed in the composite sample, and the peak intensity of the V_2_O_5_ peak is decreased as compared with V_2_O_5_. The RGO peak is not detected in the composite sample due to a lower intensity, or possibly due to the distribution of RGO over V_2_O_5_, to overlapping with the (110) reflection of V_2_O_5,_ or due to the small amount of RGO present in the composite [21].

**Figure 2:**
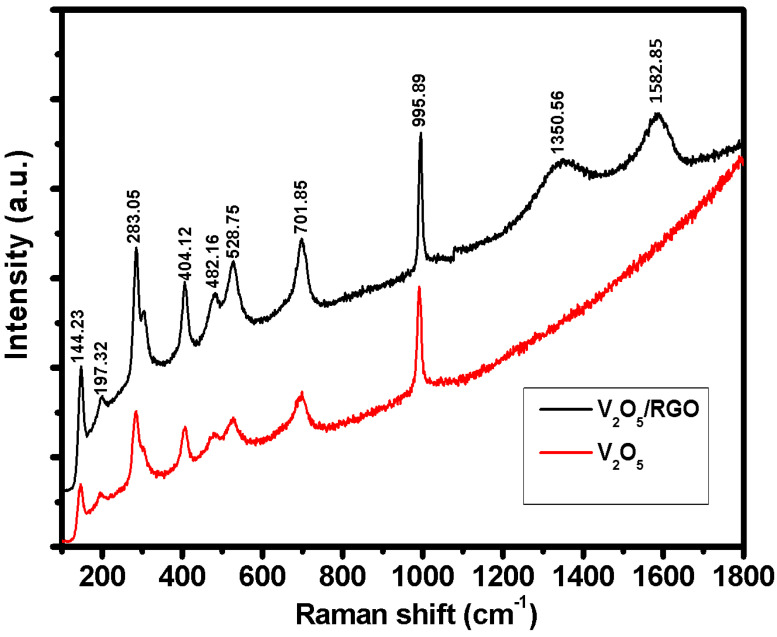
Raman spectra of V_2_O_5_ and the V_2_O_5_/RGO composite.

Raman spectroscopy was used to study the structure, symmetry and types of bonding in the V_2_O_5_ and V_2_O_5_/RGO composite. Figure 2 presents the Raman spectroscopic study of the V_2_O_5_ and V_2_O_5_/RGO composite. The V_2_O_5_ Raman spectrum exhibits multiple peaks, which are located at 144.23, 197.32, 283.05, 404.12, 482.16, and 528.75 cm^−1^. The low-frequency peak observed at 144.23 cm^−1^ corresponds to the B3g bending mode of vibration. The bending vibration of O–V–O corresponds to the peak at 197.32 cm^−1^, while the peaks at 283.05 and 404.12 cm^−1^ correspond to the oscillating Ag mode of V = atoms O. The peaks at 482.16, and 528.75 cm^−1^ are due to the vibration mode Ag of (V–O_3_–V) and ν(d3), respectively [22,23]. In the case of V_2_O_5_/RGO, two extra Raman peaks were detected at 1350.56 and 1582.85 cm^−1^, as compared to V_2_O_5_ [24]. The peak at 1350.56 cm^−1^ corresponds to the D band and the peak at 1582.85 cm^−1^ corresponds to the G band, which confirms the presence of RGO in the V_2_O_5_/RGO composite. The G band arises due to the bond stretching of sp^2^ carbon pairs in both rings and chains, and this band is associated with the optical E2g phonons at the Brillouin zone center. The D band, associated with the bending mode of aromatic rings, arises due to defects in the sample; the degree of disorder was also measured using the intensity of the D band [25].

**Figure 3:**
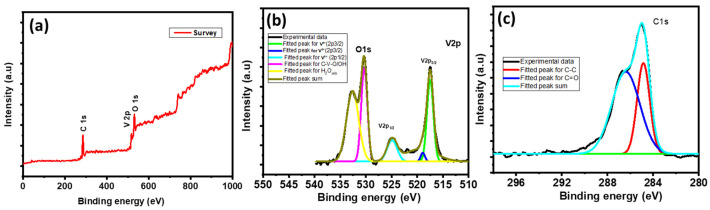
XPS spectra of V_2_O_5_/RGO composite, (**a**) a survey scan spectrum, (**b**) O1s and V2p spectra and (**c**) a C1s spectrum.

An XPS study was conducted to understand the surface composition and oxidation states of the prepared materials [26]. Figure 3 presents the results of the XPS study of the V_2_O_5_/RGO composite. The XPS survey scan spectrum of V_2_O_5_/RGO displayed in Figure 3a indicates the presence of V2p, O1s, and C1s elements. No other impurities are detected. Figure 3b presents a high-resolution spectrum of V2p and O1s. For the Vanadium 2p spectrum, binding energies of 517.44 and 524.85 eV correspond to V2p3/2 and V2p1/2, respectively, therefore vanadium is in the +5 oxidation state [27]. The Oxygen 1s spectrum is split into two major peaks, with binding energies of 530.31 and 532.63 eV; the peak at 530.31 eV corresponds to V-O bonds and the peak at 532.63 eV is associated with the presence of C-O/C=O bonds [28,29]. Figure 3c shows the C1s spectrum, with binding energies of 284.94 eV and 586.56 eV. The peak at 284.94 eV corresponds to C-C bonds with sp^3^ hybridization, while the peak at 586.56 eV is associated with contributions from both C-O and C-OH functionalities [30].

**Figure 4:**
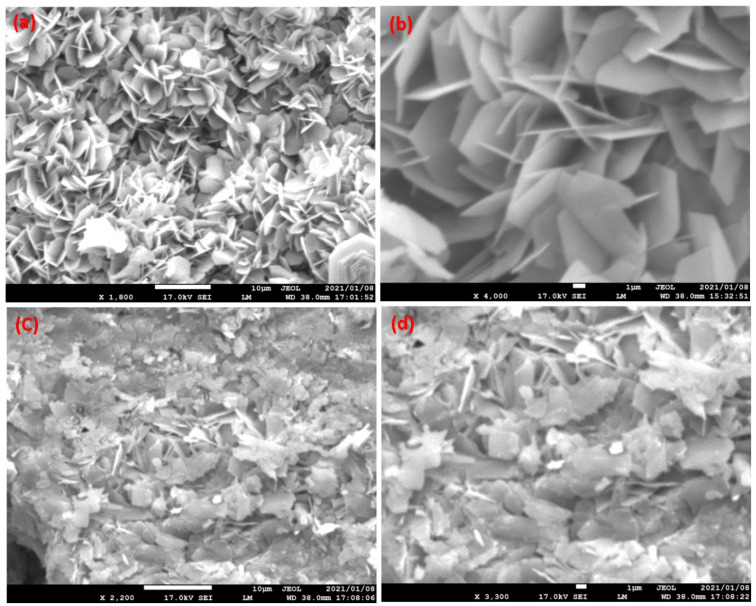
SEM images of (**a**,**b**) V_2_O_5_ and (**c**,**d**) V_2_O_5_/RGO composite, at different magnifications.

Morphology plays an important role in photocatalytic degradation activity. Prepared materials were characterized using a scanning electron microscope. Figure 4a,b present SEM images of V_2_O_5_ at different magnifications. In the SEM images, the nanoflake-shaped morphology of V_2_O_5_ is observed. The web-like structure is formed by these nanoflakes interconnecting with each other. This nanoflake-like morphology is useful for the insertion of electrolytes through the catalyst surface, providing more active surface area for redox reactions [31]. During the preparation of V_2_O_5_/RGO composite, the flake-like structure of V_2_O_5_ is disturbed, i.e., the flake-shaped structures are broken down. These V_2_O_5_/RGO nanoflake-like structures are deposited over the RGO sheet, as presented in Figure 4c,d.

**Figure 5:**
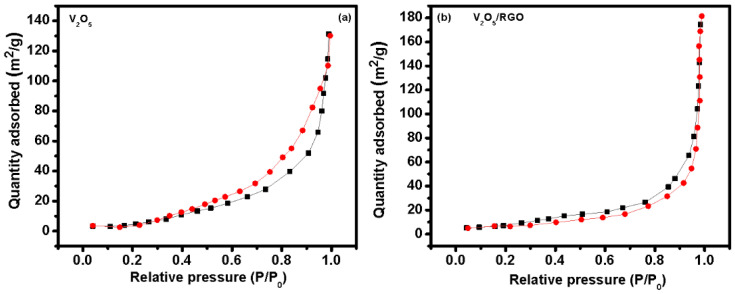
N_2_ adsorption/desorption isotherms of the (**a**) V_2_O_5_ and (**b**) V_2_O_5_/RGO composite photocatalysts. (Black colored dotted line for adsorption and red colored dotted line for desorption).

The specific surface area of the prepared catalyst materials was calculated using the Brunauer–Emmett–Teller (BET) technique. To investigate the texture properties of V_2_O_5_ and the V_2_O_5_/RGO composite, N_2_ adsorption/desorption measurements were performed, and are presented in Figure 5a,b. The isotherm profile of V_2_O_5_/RGO composite corresponds to type IV with a hysteresis loop, suggesting a porous structure. The specific surface area of the V_2_O_5_ and V_2_O_5_/RGO composite photocatalysts was found to be 31.12 and 52.17 m^2^/g, respectively. Such a large surface area of the V_2_O_5_/RGO composite photocatalyst provides more surface active sites for redox reactions, which is helpful for enhancing photocatalytic degradation efficiency [4,5].

**Figure 6:**
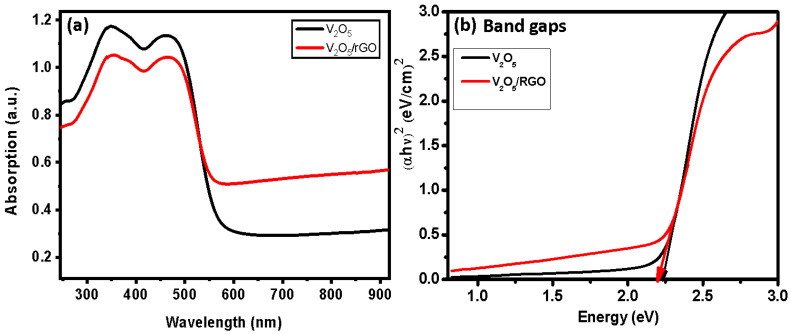
(**a**) UV-Vis absorbance spectra and (**b**) band gap plots of V_2_O_5_ and V_2_O_5_/RGO composite.

Optical properties are important when investigating photocatalytic activity. Figure 6a presents the absorption spectra of the V_2_O_5_ and V_2_O_5_/RGO photocatalysts. For both catalysts, the absorbance lies in the visible region. The V_2_O_5_ and V_2_O_5_/RGO composite photocatalysts’ absorbance edges were found at 585 and 635 nm, respectively. For the V_2_O_5_/RGO composite, the photocatalyst’s absorbance edge was shifted towards the higher wavelength side. Using the following equation, the band gap energies were calculated for V_2_O_5_ and V_2_O_5_/RGO composite, and are presented in Figure 6b [32].

αhν = A(hν − E_g_)^n^,

where hν is photon energy, A is a constant, n is order, E_g_ is band gap energy, and α is the extinction constant. For V_2_O_5_, the band gap energy was found to be 2.26 eV, while for the V_2_O_5_/RGO composite, it was 2.18 eV. The RGO in the V_2_O_5_/RGO composite was beneficial for enhancing light absorption capacity and reducing the band gap energy of V_2_O_5_, an effect attributed to the increased carrier concentrations in the valence band (VB) and conduction band (CB). In the composite, a reduction in the band gap energy is observed due to the electron traps formed in the CB, suggesting that there is a change in the electronic structure of V_2_O_5_ [4,18].

## 4. Photocatalytic Degradation Activity

**Figure 7:**
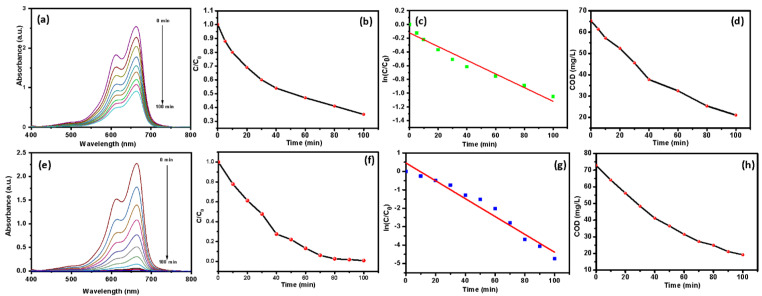
Photocatalytic degradation of MB using V_2_O_5_, (**a**) extinction spectra, (**b**) C/C_0_ vs. time, (**c**) ln(C/C_0_) vs. time and (**d**) the variation in COD values with respect to time, and photocatalytic degradation of MB using V_2_O_5_/RGO composite, (**e**) extinction spectra, (**f**) C/C_0_ vs. time, (**g**) ln(C/C_0_) vs. time and (**h**) the variation in COD values with respect to time.

A comparative study of the photocatalytic degradation of methylene blue was conducted using V_2_O_5_ and V_2_O_5_/RGO composite. Figure 7 presents the photocatalytic degradation performance of the V_2_O_5_ and V_2_O_5_/RGO composite photocatalysts under illumination, for MB dye degradation. The absorbance spectra of the MB dye, using the V_2_O_5_ photocatalyst, are presented in Figure 7a. Spectra were recorded at wavelengths ranging from 400 to 800 nm and the photocatalytic experiments were conducted for 100 min. The main extinction peak occurs at 661 nm. As reaction time elapses, the intensity of the main extinction peak decreases. Redox reactions that take place on the catalyst surface lead to the degradation of MB [33]. Using these spectra, the degradation percentage can be calculated. Using the V_2_O_5_ photocatalyst, a degradation percentage of 63 % is observed. Figure 7b displays the plot of C/C_0_ vs. time, which indicates that the concentration of MB decreases with time. The ln of this plot was used to calculate the rate constant of the reaction and to confirm its order, as presented in Figure 7c. The rate constant was found to be 0.009 min^−1^ and it was a pseudo-first-order reaction. The COD study of MB, using the V_2_O_5_ photocatalyst, is presented in Figure 7d. COD studies provide information on the concentration of oxidizable matter left in the electrolyte solution, not the concentration of the parent molecule [34]. From the plot, the COD value can be observed to decrease from 65.3 to 22.1 mg/L.

A similar experiment was conducted using the V_2_O_5_/RGO composite photocatalyst and the results are presented in Figure 7e–h. Figure 7e displays the absorbance spectra of the MB dye, using the V_2_O_5_/RGO photocatalyst. As compared to V_2_O_5_, the V_2_O_5_/RGO composite photocatalyst exhibits better photocatalytic performance. A degradation percentage of 98.85% is observed using the V_2_O_5_/RGO composite photocatalyst. The superior photocatalytic performance of the V_2_O_5_/rGO composite photocatalyst is attributed to enhanced light absorption capacity, effective charge transfer, and minimum charge recombination, etc. In addition, a large number of active sites are available for redox reactions as compared to the V_2_O_5_ photocatalyst [11,16,35]. Active sites are responsible for the generation of highly reactive hydroxyl and superoxide radicals that react with organic impurities and mineralize them into CO_2_ and H_2_O. Figure 7f presents the plot of C/C_0_ vs. time. A trend similar to that observed with V_2_O_5_ was observed in the case of the V_2_O_5_/RGO composite photocatalyst, i.e., the concentration decreased with respect to time. Figure 7g displays the plot of ln(C/C_0_) vs. time. By applying a linear fit to this plot, the value of the rate constant may be obtained; a reaction rate constant of 0.048 min^−1^ is detected, which is higher than that of V_2_O_5_. In addition, the R2 (linear coefficient value) value is 0.96, which is close to unity; therefore, it follows a pseudo-first-order reaction. Figure 7h shows the plot of COD values vs. time and shows that the COD values decreased with respect to time. COD values decreased from 72.8 to 19.2 mg/L.

### Reaction Mechanism

Based on the above discussion, the mechanism of the photocatalytic degradation of MB dye using the V_2_O_5_/RGO photocatalyst is discussed. This consists of the excitation of the catalyst material by light, the generation of radicals, and the interaction of radicals with the organic compounds. Upon illumination of the V_2_O_5_/RGO catalyst, the electrons in the valence band absorb sufficient amounts of energy and are excited to the conduction band, which simultaneously creates holes in the valence band. Excited conduction band electrons can react with dissolved oxygen molecules and generate superoxide radical anions [11,36]. These superoxide radicals react with water or hydroxyl ions, thus generating hydroperoxy radicals (HOO•). Holes in the valence band react with water molecules to produce hydroxyl radicals. These superoxide and hydroxyl radicals are highly reactive in nature [37,38]. They react with dye molecules and transform them into non-toxic compounds. Here, the role of RGO is to reduce recombination of photogenerated charge carriers, promote effective charge transfer and enhance photocatalytic efficiency. Finally, the hydroxyl radicals, which are able to oxidize and mineralize MB molecules, result in the production of different species, such as carbon dioxide and water, as well as other intermediates of decomposition at much lower concentrations.

## 5. Conclusions

The V_2_O_5_ and V_2_O_5_/RGO composite photocatalysts were prepared using a simple, chemical, and cost-effective hydrothermal method. The prepared photocatalyst materials were characterized using different characterization techniques. XRD study confirms the orthorhombic crystal structure of V_2_O_5._ XPS study shows the vanadium to be in the +5 oxidation state and the carbon to be in the sp^3^ hybridization state. UV-Vis spectroscopy shows that the light absorption capacity of V_2_O_5_ is improved and the band gap energy is decreased, when forming a composite with RGO; the photocatalytic properties of the V_2_O_5_/RGO composite exhibit an enhanced photocatalytic performance, relative to V_2_O_5_. The enhancement in the photocatalytic properties of the V_2_O_5_/RGO composite is attributed to the presence of RGO, which provides efficient separation, fast transfer and minimizes recombination of photogenerated charge carriers.

## Data Availability

Data is contained within the article.
